# The DA antagonist tiapride impairs context-related extinction learning in a novel context without affecting renewal

**DOI:** 10.3389/fnbeh.2015.00238

**Published:** 2015-09-03

**Authors:** Silke Lissek, Benjamin Glaubitz, Oliver T. Wolf, Martin Tegenthoff

**Affiliations:** ^1^Department of Neurology, BG University Hospital Bergmannsheil, Ruhr-University BochumBochum, Germany; ^2^Department of Cognitive Psychology, Faculty of Psychology, Institute for Cognitive Neuroscience, Ruhr-University BochumBochum, Germany

**Keywords:** context-related extinction learning, renewal effect, fMRI, dopamine, tiapride, hippocampus, prefrontal cortex

## Abstract

Renewal describes the recovery of an extinguished response if recall is tested in a context different from the extinction context. Behavioral studies demonstrated that attention to relevant context strengthens renewal. Neurotransmitters mediating attention and learning such as the dopaminergic (DA) system presumably modulate extinction learning and renewal. However, the role of DA for non-fear-based extinction learning and renewal in humans has not yet been investigated. This fMRI study investigated effects of DA-antagonism upon context-related extinction in a predictive learning task in which extinction occurred either in a novel (ABA) or an unchanged (AAA) context. The tiapride-treated group (TIA) showed significantly impaired ABA extinction learning and a significant within-group difference between ABA and AAA extinction, compared to placebo (PLAC). Groups did not differ in their level of ABA renewal. In ABA extinction, TIA showed reduced activation in dlPFC and OFC, hippocampus, and temporal regions. Across groups, activation in PFC and hippocampus correlated negatively with ABA extinction errors. Results suggest that in context-related extinction learning DA in PFC and hippocampus is involved in readjusting the cue-outcome relationship in the presence of a novel context. However, relating context to the appropriate association during recall does not appear to rely exclusively on DA signaling.

## Introduction

The renewal effect of extinction describes the recovery of an extinguished response when extinction learning has been performed in a context different from that present during extinction recall (Bouton and Bolles, 1979). Thus, it highlights the context-dependency of extinction. In a recent imaging study in humans, we demonstrated that renewal is mediated by hippocampus and ventromedial prefrontal cortex (vmPFC) in concert (Lissek et al., 2013). During extinction learning, hippocampal activation is more pronounced in participants who later exhibit renewal than in those who do not, suggesting that their encoding of context is more effective (Lissek et al., 2013). These results are in line with previous findings in human fear extinction that associated hippocampus and vmPFC with context processing (Kalisch et al., 2006; Milad et al., 2007). Behavioral studies of renewal showed that modulation of attention is guided by stimulus relevance (Uengoer and Lachnit, 2012), and that allocation of attention can be controlled by contextual stimuli (Uengoer et al., 2013). Also on the behavioral level, it has been demonstrated that a task designed to focus attention upon context actually strengthens renewal in participants who have implicitly learned that context is relevant (Lucke et al., 2013). This finding is consistent with the notion that the strength of context-specific learning depends on the amount of attention paid to context stimuli (Rosas and Callejas-Aguilera, 2006). In consequence, it is conceivable that the renewal effect is dependent on attentional and encoding processes that occur during extinction learning and thus may be mediated by neurotransmitter systems involved in learning and attention, such as the noradrenergic and dopaminergic systems (Lauzon et al., 2009). Recent studies in humans and rats meanwhile showed that while stimulation of the noradrenergic system actually enhanced extinction learning, it had no impact upon the strength of the renewal effect (André et al., 2015; Lissek et al., 2015).

A general role for dopamine (DA) in Pavlovian and instrumental learning is well-established (Schultz et al., 1997; Schultz, 1998). DA is involved in both the learning and the attentional aspects of conditioning (El-Ghundi et al., 2007), directing attention to salient and novel stimuli, and delivering a teaching and reward signal during associative learning (Reynolds et al., 2001). DA receptor antagonism in prefrontal cortex (PFC) can affect performance in various aspects of tasks that require attention, such as set-shifting and reversal of a learned response (Boulougouris and Tsaltas, 2008). In a number of animal studies, participation of the dopaminergic system in extinction learning was demonstrated for D1 and D2 receptors. In rats, D1 agonists affected fear extinction learning (Fiorenza et al., 2012; Rey et al., 2014), while D1 antagonists (SCH23390) decreased renewal of a Pavlovian-conditioned response (alcohol-seeking) (Sciascia et al., 2014), affected contextual fear extinction (Fiorenza et al., 2012) and prolonged extinction of place preference (Fricks-Gleason et al., 2012). Mice deficient in D1 receptors showed delayed fear extinction (El-Ghundi et al., 2001). Moreover, D1 receptor antagonism modulated performance in a task of contextual control of response conflict (Haddon and Killcross, 2011). For fear extinction, in particular infralimbic D2 receptors appear to be necessary, since local inactivation of infralimbic cortex in rats impaired extinction learning (Mueller et al., 2010). D2 antagonism accelerated fear extinction in mice (Ponnusamy et al., 2005; Dubrovina and Zinov'eva, 2010), while D2 agonism blocked fear extinction in rats (Nader and LeDoux, 1999).

While human data on effects of manipulating the dopaminergic system during extinction learning are lacking, there are studies reporting improving effects of DA-agonists upon other forms of human learning (Breitenstein et al., 2004; Flöel et al., 2005; Breitenstein et al., 2006). Moreover, a recent study on fear extinction in humans demonstrated that the dopamine precursor L-Dopa, administered after extinction, made extinction memories context-independent and thus reduced the return (renewal) of fear (Haaker et al., 2013). Animal studies also implicated the dopaminergic system in renewal. Administration of a DA1 antagonist (SCH23390) before extinction recall prevented renewal of an extinguished instrumental response (Hamlin et al., 2006). Pretreatment with D1 and D2 receptor antagonists attenuated context-induced renewal of cocaine seeking (Crombag et al., 2002) or sucrose seeking (Rauhut et al., 2010) in rats. Taken together, studies in animals and humans have delivered ample evidence for the involvement of the dopaminergic system in fear extinction. However, its function for contextual extinction learning and renewal without a fear component has not yet been investigated in humans.

Conceivably, the relevance of the dopaminergic system for fear extinction learning may be associated with its functions in prefrontal and hippocampal regions during learning and processing of context. Both areas are target regions for dopaminergic influences: expression of D1 and D2 receptors was reported for prefrontal cortex of rodents (Vincent et al., 1995) and for hippocampus of rodents and primates (Camps et al., 1990). In humans, mRNA for all types of dopaminergic receptors is expressed in prefrontal cortex (Meador-Woodruff et al., 1996). In human hippocampus, a moderate to high expression of D2 (Hurd et al., 2001), and a low to moderate expression of D3 receptor mRNA (Suzuki et al., 1998) was observed. In general, dopamine in the prefrontal cortex may be important for extinction by gating cognitive and behavioral flexibility (Abraham et al., 2014). Studies in rats and mice demonstrated that dopaminergic modulation of prefrontal regions can also affect attentional performance and working memory (Granon et al., 2000; Chudasama and Robbins, 2004; Glickstein et al., 2005). Accordingly, local infusions of a D1/D2 receptor antagonist into prelimbic cortex of the rat caused impairments in adaptations of instrumental responses to changes in contingency, suggesting a role for this region in action-outcome associations (Naneix et al., 2009). Dopamine-mediated activity in human ventromedial PFC is involved in evaluating potential choices when learning to guide reinforcement-based decisions (Jocham et al., 2011). DA release in mOFC, vmPFC as well as dACC is important in reinforcement learning in the human brain, as a PET study measuring dopamine during a reward learning task demonstrated (Vrieze et al., 2013). DA infusions into vmPFC of rats influenced outcome sensitivity (Hitchcott et al., 2007), suggesting that the dopaminergic system in vmPFC has a role in response choices. In line with these findings, local infusion of both D1 or D2 antagonists into rat vmPFC impaired fear extinction (Mueller et al., 2010; Fiorenza et al., 2012).

Regarding dopaminergic influences in hippocampus, recent evidence indicates that hippocampal dopamine has a crucial role in memory formation, promoting memory for episodes that are novel and rewarding as well as building memory representations suited to guide later behavioral decisions (Shohamy and Adcock, 2010). Hippocampal D2 receptor activity was found correlated with memory function in humans (Takahashi et al., 2008), while D1 receptor modulation in rat hippocampus has been shown to affect fear extinction (Fiorenza et al., 2012). Furthermore, a PET study demonstrated that D1 receptor activity in hippocampus was positively linked to executive performance and speed (Karlsson et al., 2011).

In the present study, we aimed at investigating the role of the dopaminergic system in humans for context-related extinction learning without a fear component as well as for the renewal effect. We used an associative learning task in which participants were required to learn relations between cues and outcomes presented in particular contexts, which were reversed during the extinction learning phase. This predictive learning task (Ungör and Lachnit, 2006), which we already used in previous studies (Lissek et al., 2013, 2015) features an ABA design suited to reliably evoke a renewal effect, combined with a control AAA condition that does not evoke renewal. We treated healthy participants with a single dose of the D2/D3 antagonist tiapride prior to an extinction learning session of previously acquired associations.

We hypothesized that the DA-antagonist, compared to placebo, would impair extinction learning performance. In addition, we assumed that due to weak extinction associations in DA-antagonist treated participants, a greater number of acquisition associations would be recovered during extinction recall not only in ABA but also in the AAA condition, an outcome that reflects a reduction in actual ABA renewal. Moreover, we expected a concurrent reduction in activation of brain regions participating in extinction learning and attentional processing, such as prefrontal cortex and hippocampus.

## Materials and methods

### Participants

Forty healthy right-handed volunteers (19 females, 21 males), mean age 25.60 years ± 5.16 years st.dev., range 20–31 years, without a history of neurological disorders (questionnaire, self-report), participated in this study. The participants received a monetary compensation for their participation (in the amount of € 60). Participants were randomly assigned to the experimental tiapride (TIA) and placebo control (PLAC) groups. Mean age within the groups was 25.68 years ± 4.92 st.dev., range 20–36 years in TIA and 24.88 years ± 3.20 st.dev., range 20–31 years in PLAC. Participants were assigned to the groups showing (REN) and not showing renewal (NOREN) according to the procedure described in “Behavioral data analysis.”

### Ethics statement

All subjects participated in this study after giving written informed consent. The protocol was approved by the Ethics Committee of the Ruhr-University Bochum. The study conforms to the Code of Ethics of the World Medical Association (Declaration of Helsinki). Prior to the experiments, participants received handouts informing them about the fMRI procedures and the DA-antagonist Tiapride.

### Predictive learning task

The predictive learning task that we used in this study was originally developed by Ungör and Lachnit (2006) to explore the context-dependency of extinction learning. Its efficiency in evoking a renewal effect was demonstrated in several behavioral studies using this specific design (Rosas and Callejas-Aguilera, 2006; Ungör and Lachnit, 2006; Üngör and Lachnit, 2008; Nelson and Callejas-Aguilera, 2007; Lucke et al., 2013). We adapted this task for use in an fMRI setting and already used it in previous fMRI studies (Lissek et al., 2013, 2015).

**Figure 1:**
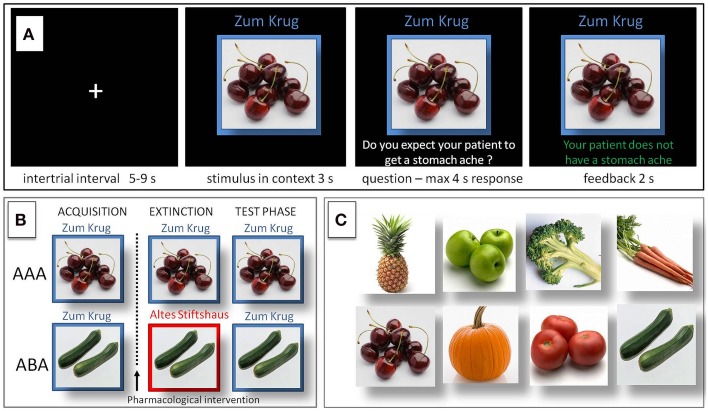
**Predictive learning task. (A)** Example of a trial during acquisition of the task. Participants learned to predict whether certain kinds of food, eaten in a certain restaurant, would cause a stomach ache or not. After an intertrial interval of 5–9 s the stimulus was presented in its context for 3 s, then a question was superimposed on the screen “Do you expect your patient to get a stomach ache?” for maximum 4 s response time. Feedback was shown for 2 s, providing the correct answer, e.g., “The patient does not have a stomach ache.” **(B)** Design of the predictive learning task. In condition AAA, extinction occurs in the same context as acquisition. In condition ABA, extinction occurs in a context different from that during acquisition. In both conditions, the final test for the renewal effect is performed in the context of acquisition. **(C)** Food images used as stimuli.

In the predictive learning task, participants were asked to put themselves in the position of a physician and predict whether various articles of food served in different restaurants would lead to the aversive consequence of a stomach ache in their patient. The learning process consisted of the three successive phases of (a) acquisition of associations, (b) extinction, and (c) recall phase (see Figure 1). During the acquisition phase (80 trials) participants learned to associate an article of food with a specific consequence. In each trial one of eight stimuli (vegetables or fruits) was presented to the participant in one of two different contexts (indicated by the restaurant names “Zum Krug” (The Mug) and “Altes Stiftshaus” (The Dome) and a frame in either red or blue color). The stimulus in its context was first presented for 3 s, then a question asking whether the patient will develop a stomach-ache was superimposed, with the response options “Yes” or “No.” Response time was 4 s, participants responded by pressing the respective button on an fMRI-ready keyboard (Lumitouch, Photon Control Inc. Canada). After the response, or in case of a missing response after expiration of the response time, a feedback with the correct answer was displayed for 2 s, i.e., “The patient has a stomach ache” or “The patient does not have a stomach ache.” The actual response of the participant was not commented upon. The food stimuli were presented in randomized order, each stimulus was presented 10 times. Four stimuli were presented per context. Stimuli were counterbalanced with regard to their causing the aversive consequence of a stomach ache, with two stimuli per context causing stomach ache during acquisition, while the other two did not.

During the extinction phase (80 trials), half of the stimuli were presented in the same context as during acquisition (condition AAA—no context change—40 trials) and the other half in the other context (condition ABA—context change—40 trials) in randomized order. In addition, stimuli were subdivided into two types: for actual “extinction stimuli,” the consequence changed and the new consequence had to be learned, for “distractor stimuli,” which were introduced in order to make overall learning more difficult, the consequence remained unchanged. Per context we used two extinction stimuli and two distractor stimuli. In all other respects, trials were identical to those during acquisition.

During the recall phase (40 trials), all stimuli were presented once again in the context of acquisition (five presentations per stimulus). With the exception that during the recall phase no feedback with the correct response was given, trials were identical to those during acquisition.

### Procedure

In a first fMRI session, participants passed the acquisition phase of the predictive learning task. Immediately after this session, the dopaminergic antagonist tiapride was administered orally in a single dose of 100 mg. Control participants received an identical-looking placebo. One hundred and twenty minutes after administration of the drug/placebo, in accordance with the pharmacokinetic profile of tiapride with peak plasma concentrations achieved around this time point (Rey et al., 1982; Norman et al., 1987), the second fMRI session was performed, which comprised the extinction learning phase and the extinction recall phase. Tiapride is a selective antagonist of D2 and D3 dopamine receptors (Dose and Lange, 2000), which has previously been shown to impair motor learning in humans (Lissek et al., 2014), as well as taste (Mediavilla et al., 2012) and place (Hurtado et al., 2014) aversion learning in rats. A study in non-human primates showed that tiapride down-regulated dopaminergic D1-receptors in prefrontal cortex, indicating that D2 receptor antagonism may have an impact upon D1 receptors too (Lidow et al., 1997).

### Imaging data acquisition

Functional and structural brain scans were acquired using a whole-body 3T scanner (Philips Achieva 3.0 T X-Series, Philips, The Netherlands) with a 32-channel SENSE head coil. Blood-oxygen level dependent (BOLD) contrast images were obtained with a dynamic T2^*^ weighted gradient echo EPI sequence using SENSE (TR 3200 ms, TE 35 ms, flip angle 90°, field of view 224 mm, slice thickness 3.0 mm, voxel size 2.0 × 2.0 × 3.0 mm). We acquired 45 transaxial slices parallel to the anterior commissure—posterior commissure (AC-PC) line which covered the whole brain. High resolution structural brain scans of each participant were acquired using an isotropic T1 TFE sequence (field of view 240 mm, slice thickness 1.0 mm, voxel size 1 × 1 × 1 mm) with 220 transversally oriented slices covering the whole brain.

The task was presented to the participants via fMRI-ready LCD-goggles (Visuastim Digital, Resonance Technology Inc., Northridge, CA, USA) connected to a laptop which ran specific software programmed in Matlab (Mathworks, Natick, MA, USA). Responses were given by means of an fMRI-ready keyboard (Lumitouch response pad, Photon Control Inc., Canada).

### Imaging data analysis

For preprocessing and statistical analysis of fMRI data we used the software Statistical Parametric Mapping (SPM), Version 8 (Wellcome Department of Cognitive Neurology, London, UK), implemented in Matlab R2008a (Mathworks, Natick, MA, USA). Three dummy scans, during which BOLD signal reached steady state, preceded the actual data acquisition of each session, thus preprocessing started with the first acquired volume. Preprocessing on single subject level consisted of the following steps: slice timing correction to account for time differences due to multislice image acquisition; realignment of all volumes to the first volume for motion correction; spatial normalization into standard stereotactic coordinates with 2 × 2 × 2 mm^3^ using an EPI template of the Montreal Neurological Institute (MNI), smoothing with a 6 mm full-width half-maximum (FWHM) kernel, in accordance with the standard SPM procedure. The acceptable limit for head motion was 2 mm for translational movements and 0.5° for rotational movements.

In a first level single subject analysis, we calculated activation during extinction and recall phases in the conditions ABA and AAA, respectively. The contrasts were calculated within a combined anatomically defined mask which was constructed using the software MARINA (BION Bender Institute of Neuroimaging, University of Giessen, Germany) (Walter et al., 2003). The mask was centered around *a priori* regions of interest, containing prefrontal cortex, hippocampus, amygdala, insula, and temporal lobe. All data contained in this combined mask were analyzed together in a single analysis. We used an event-related design, modeling the events of each trial (stimulus and questions presentation, feedback presentation) using distinct stick functions convolved with the default HRF in SPM, with our analysis based on the stimulus presentation phase of each trial. The contrast images from these analyses were entered into second-level random-effects analyses to calculate in one-sample tests the activation patterns of the experimental and control groups for the different contrasts, using a threshold of *p* < 0.001 FWE-corrected on cluster level. Moreover, we calculated two-sample tests to directly investigate in which regions the experimental group showed differential activation compared to controls, using a threshold of *p* < 0.05 FWE-corrected on cluster level for the reported activations.

For additional analyses in which we correlated BOLD signal changes to performance data, we extracted the mean signal intensities (in arbitrary units) of activated clusters derived from the two-sample tests comparing the TIA and PLAC groups, using the MarsBar toolbox (Brett et al., 2002) in SPM 8.

### Behavioral data analysis

For all three learning phases, log files were written that contained information on response latency, response type, and correctness of response. In acquisition, a response giving the wrong prediction was considered an error. Again, in extinction, a response giving the wrong prediction was considered an error. Thus, for extinction trials with a consequence change, a response that was correct during acquisition was considered an error during extinction. For distractor trials (no consequence change), the correct response remained the same as during acquisition.

For calculation of the renewal effect, only responses to stimuli with consequence change (extinction stimuli) during the recall phase were analyzed. The behavioral renewal effect in the predictive learning task is supposed to occur only in the condition ABA, in which extinction is performed in a context different from the context present during acquisition and recall phase. During the ABA recall phase, a renewal response occurs if the answer reports the association that was correct during acquisition, but wrong during extinction (e.g., if in acquisition in context A cherries cause stomach ache, and in extinction in context B they do not cause stomach ache any more, then a renewal effect response during recall in context A states that cherries cause stomach ache.). During the AAA recall phase, a response that reports an association that was correct during acquisition is considered an error, for since extinction occurred in an identical context, recalling the most recent association would be correct. Statistical analyses were performed using the IBM SPSS Statistics for Windows software package, version 22.0 (Armonk, NY; IBM Corp.). We used one-tailed *t*-tests to test our directional hypotheses regarding performance impairments following the experimental treatment.

In previous studies using the predictive learning task we found that a considerable portion (about 40%) of the participants did not exhibit the renewal effect. This is a typical finding that also appears in this type of task outside an fMRI setting (Lissek et al., 2013). For further evaluation of their behavioral data, participants were grouped according to whether they showed renewal (REN) or did not show renewal (NOREN). Group assignment was based on participants' performance during the recall phase in those trials designed to evoke renewal (i.e., the ABA trials with consequence change). All participants who never showed a renewal effect (0% renewal responses) were assigned to the NOREN group, and all participants who showed a renewal effect (30–100% renewal responses) were assigned to the REN group.

## Results

### Behavioral results

#### Acquisition

We observed no significant differences in acquisition performance (pre-treatment) between the groups: *t*_(38)_ = 0.042 *p* = 0.967 two-tailed (percent errors mean ± SE: TIA 16.50% ± 3.37, PLAC 16.69% ± 2.88).

#### Extinction

**Figure 2:**
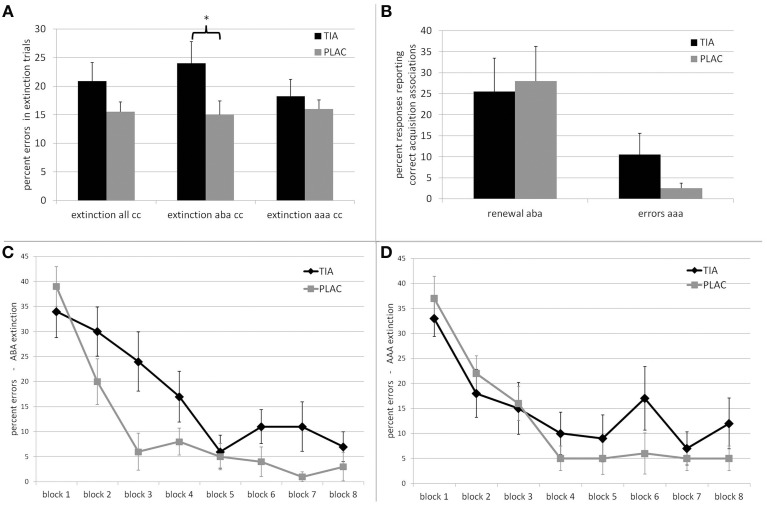
**Top:** Behavioral performance of the TIA (black) and PLAC (gray) groups. **(A)** Percentage of errors in extinction learning for trials with a consequence change (cc), for all trials, ABA, and AAA trials. ^*^The difference is significant at *p* < 0.05. **(B)** Percentage of responses in extinction recall that report associations correct during acquisition, that is responses which constitute renewal responses in ABA trials and errors in AAA trials. **Bottom:** Learning curve for **(C)** ABA extinction and **(D)** AAA extinction. Error bars denote standard errors.

As hypothesized, we observed extinction learning impairments in the TIA group. For overall extinction learning performance, there was a trend toward a significant difference between groups regarding errors in trials with a consequence change [*t*_(38)_ = 1.453 *p* = 0.078; percent errors mean ± SE: TIA 20.87% ± 3.25; PLAC 15.50% ± 1.75]. When considering only extinction learning in a novel context (ABA condition), the TIA group was significantly impaired compared to PLAC [*t*_(38)_ = 1.989 *p* = 0.027; TIA 24.00% ± 3.81; PLAC 15.00% ± 2.43], while there was no significant difference in AAA extinction learning between groups [*t*_(38)_ = 0.673 *p* = 0.252; TIA 18.25% ± 2.93; PLAC 16.00% ± 1.59—all *t*-tests one-tailed]. (See Figure 2A) Moreover, within the TIA group, we found a significant difference between extinction learning performance in the ABA and the AAA conditions [*t*_(19)_ = 2.498 *p* = 0.022], which is absent in the PLAC group [*t*_(19)_ = 0.462 *p* = 0.649].

Regarding error rates in distractor trials (no consequence change), we observed no significant differences between TIA and PLAC [*t*_(39)_ = 0.522 *p* = 0.605; percent errors mean ± SE: TIA 12.0% ± 3.97; PLAC 9.5% ± 2.69], suggesting a comparable memory for associations learned during acquisition.

#### Learning curve

In order to evaluate the groups' learning progress, we divided the extinction session into eight blocks with 10 trials each and calculated the percentage of extinction errors in ABA and AAA separately for each of these blocks. (See Figures 2C,D) For ABA extinction learning, a repeated measures ANOVA showed a significant main effect of the repeated measures factor learning block [*F*_(7, 39)_ = 29.998 *p* = 0.000] upon error rates and a significant interaction of learning block^*^treatment [*F*_(7, 39)_ = 2.794 *p* = 0.008], indicating that learning progressed differently in TIA and PLAC. The factor treatment showed a trend toward a significant main effect upon the overall progress of learning [*F*_(1, 39)_ = 3.169 *p* = 0.083]. For further analyses, we grouped the 8 blocks into three phases: initial exposure to changed stimulus-outcome contingencies (1st block), early extinction learning (blocks 2–5) and late extinction learning (blocks 6–8). While TIA and PLAC showed similar error rates during initial exposure to the changed stimulus-outcome contingencies [*t*_(38)_ = −0.760 *p* = 0.226; TIA 34.0% ± 5.25; PLAC 39% ± 3.96], during the following early extinction learning phase the TIA group made significantly more errors than the PLAC group [*t*_(38)_ = 2.112 *p* = 0.020; TIA 19.25% ± 3.81; PLAC 9.75% ± 2.39]. In later extinction learning, the performance difference persisted [*t*_(38)_ = 1.919 *p* = 0.031; TIA 9.67% ± 3.33; PLAC 2.67% ± 1.48] (all *t*-tests one-tailed). Despite this slower learning progress, the TIA group showed extinction learning also in the ABA condition, with their rate of correct responses exceeding 90% in the final blocks.

For AAA extinction, an ANOVA with repeated measures yielded a significant main effect of the repeated factor learning block [*F*_(7, 39)_ = 18.597 *p* = 0.000], while the interaction learning block^*^treatment [*F*_(7, 39)_ = 1.327 *p* = 0.237] and the factor treatment [*F*_(1, 39)_ = 0.536 *p* = 0.468] showed no significant effect. In summary these results indicate a comparable learning progress in both groups over the course of AAA extinction learning.

#### Renewal

In both groups, participants who showed or did not show the renewal effect were equally distributed (TIA: χ^2^ = 0.800; *p* = 0.371; REN 40% NOREN 60%; PLAC: χ^2^ = 0.000; *p* = 1.00; REN 50% NOREN 50%). Renewal rates in REN participants ranged from 30 to 100% in both TIA and PLAC groups.

The dopamine antagonist tiapride had no effect upon contextual extinction retrieval: TIA and PLAC did not differ regarding the strength of the renewal effect (i.e., the percentage of renewal responses in the ABA condition): *t*_(38)_ = −0.218 *p* = 0.418 one-tailed (mean ± SE: TIA 25.50% ± 7.929; PLAC 28.00% ± 8.29). When comparing only those participants who actually showed a renewal effect, we again observed no significant difference between groups: *t*_(16)_ = 0.433 *p* = 0.670 two-tailed. (TIA 70.83% ± 8.76; PLAC 68.33% ± 10.07) (See Figure 2B).

On the other hand, TIA participants showed a trend toward impairment in retrieving the proper answer for trials in which extinction was performed in the acquisition context (AAA condition), which in the test phase required to retrieve the most recently acquired, altered association: *t*_(38)_ = 1.539 *p* = 0.066 one-tailed (mean percent errors in AAA: TIA 10.5% ± 5.05 s.e.; PLAC 2.5% ± 1.23 s.e.).

### Imaging results

#### Activation patterns of TIA and PLAC during extinction learning and recall

##### Extinction learning

**Figure 3:**
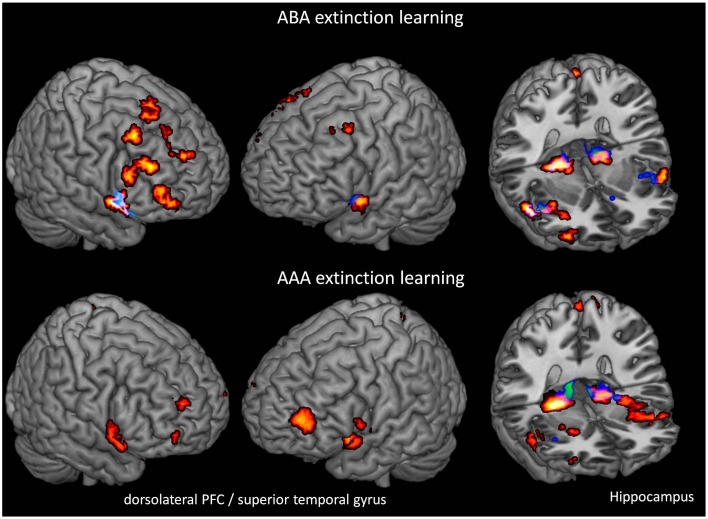
**Overlays of activation patterns in the PLAC (yellow-red) and the TIA (blue-green) group during the ABA and AAA conditions of extinction learning**. The TIA group exhibits reduced activation in various regions, including dorsolateral prefrontal cortex and hippocampus in both ABA and AAA conditions. (one-sample *t*-tests *p* < 0.001 FWE-corrected on cluster level, minimum cluster size *k* = 10).

We performed one-sample *t*-tests of TIA and PLAC during extinction learning in the ABA and AAA conditions, respectively. During extinction learning in both the ABA and AAA conditions, both groups show activation in hippocampus, fusiform gyrus, lingual gyrus, and insula. In contrast to PLAC, however, the TIA group shows no activation in dlPFC, lateral OFC, and superior temporal gyrus. The difference in dlPFC activation is particularly prominent for the ABA condition, where the PLAC group activates a number of clusters in bilateral BA 8, 9, and 46, while there is no dlPFC activation in TIA (see Table 1 and Figure 3).

**Table 1:** **One-sample tests—activated regions in TIA and PLAC during Extinction learning ***p*** < 0.001 FWE-corrected, ***k*** = 10**.

**Brain region**	**BA**	**Hem**	**EXTINCTION ABA**						**EXTINCTION AAA**					
			**TIA**			**PLAC**			**TIA**			**PLAC**		
			**MNI x y z**	***t*-value**	**voxel**	**MNI x y z**	***t*-value**	**voxel**	**MNI x y z**	***t*-value**	**voxel**	**MNI x y z**	***t*-value**	**voxel**
dlPFC	46	R				46 40 28	8.93	92				22 50 20	7.52	46
	9	R				20 56 30	7.67	21						
						22 42 40	7.33	31						
	8	R				42 22 48	9.34	71						
		L				−30 18 46	6.40	17						
						−42 14 50	6.33	12						
OFC lateral	10	R				46 50 10	7.42	65				38 56 0	6.14	10
		L										−38 54 14	9.64	138
OFC orbital	47	R	50 14 0	8.81	84				44 24 −12	6.73	45			
		L										−46 16 −8	8.83	82
Hippocampus		R				18 −30 −4	9.01	23				20 −28 −6	12.91	73
		L	−18 −30 −6	9.23	33				−28 −22 −12	7.19	23	−22 −32 −6	8.62	37
Superior temporal gyrus	22	R				54 20 −8	8.79	142				50 12 −6	7.27	86
		L				−56 10 −6	7.49	60				−56 12 −6	6.08	82
Temporal pole		R										54 18 −10	7.22	37
Insula		R	30 20 −14	8.48	141	42 18 −6	7.99	128	28 20 −14	6.83	13			
		L	−46 8 −8	6.82	27							−32 −18 4	9.55	130
Fusiform gyrus		R	32 −52 −14	10.57	178	32 −36 −24	10.37	18						
		L				−38 −36 −24	9.19	30						
	37	R				36 −50 −16	7.43	32	36 −52 −14	7.01	22	36 −52 −14	7.30	50
		L	−30 −48 −16	12.88	55	−22 −48 −16	7.75	47	−28 −46 −18	7.11	10	−34 −40 −22	6.86	29
Lingual gyrus		R	20 −50 −6	8.68	98				12 −42 −4	6.20	67			
		L	− 24 −48 −8	6.73	44	−16 −50 −8	7.30	32	−8 −35 −4	6.56	13	−22 −48 −16	7.02	17
Parahippocampal gyrus	27	R	20 −35 −14	7.42	106									
		L										−16 −24 2	8.45	27
Posterior cingulate	30	R							6 40 6	9.45	13			

##### Recall

During extinction recall, both groups show activation in fusiform gyrus, lingual gyrus, cingulate gyrus, insula, and dlPFC, as well as in hippocampus, which, however, is not activated in PLAC during AAA recall. In addition, PLAC, in contrast to TIA, shows no activation in lateral OFC and the temporal pole. TIA, in contrast to PLAC, does not activate regions in parahippocampal gyrus (see Table 2).

**Table 2:** **One-sample tests—activated regions in TIA and PLAC during Extinction recall ***p*** < 0.001 FWE-corrected, ***k*** = 10**.

**Brain region**	**BA**	**Hem**	**RECALL ABA**						**RECALL AAA**					
			**TIA**			**PLAC**			**TIA**			**PLAC**		
			**MNI x y z**	***t*-value**	**voxel**	**MNI x y z**	***t*-value**	**voxel**	**MNI x y z**	***t*-value**	**voxel**	**MNI x y z**	***t*-value**	**voxel**
dlPFC	46	R	46 34 26	9.98	11									
	9	R	48 12 40	8.86	63							38 44 34	7.15	13
		L	−52 14 42	9.44	38	−38 26 32	7.22	22				−56 8 36	6.63	20
	8	R							54 14 44	8.25	31	52 10 38	6.71	25
		L							−50 10 44	6.74	17			
OFC lateral	10	R	38 52 20	6.94	33				30 52 10	7.42	13			
		L	−32 44 10	10.19	90				−32 44 24	7.80	32			
OFC orbital	47	R	56 14 −2	8.17	31	32 24 −6	6.49	63	52 20 0	6.21	30			
		L	−44 14 −6	8.99	39									
														
Hippocampus		R	22 −26 −8	9.18	14	24 −28 −6	7.89	21						
		L							−22 −22 −10	8.81	11			
Temporal pole		R	50 14 −12	8.99	22									
		L	−54 14 −8	6.51	67									
Insula		R	36 22 −2	12.44	252	34 18 −2	8.71	111	32 22 −8	10.49	119	42 20 −6	6.41	15
		L	−46 2 2	9.10	81	−40 12 −2	6.83	55	−32 18 6	9.68	66			
						−38 −2 10	7.62	30						
														
Fusiform gyrus	20	R	32 −34 −26	7.96	21	32 −52 −14	7.88	127	28 −52 −14	6.49	41	28 −50 −14	9.66	132
		L										−38 −40 −22	7.95	45
	37	L	−24 −48 −16	8.45	76	−26 −50 −12	7.89	25						
	19	R	28 −52 −10	10.35	111									
Lingual gyrus		R	16 −44 −2	6.85	17	20 −44 −8	6.45	19	18 −46 −12	6.93	7	16 −48 −8	6.85	35
		L	−20 −48 −6	9.16	97	−18 −44 −10	7.03	10				−20 −50 −10	6.51	10
Parahippocampal gyrus	27	R				22 −40 −8	7.20	47				18 −42 −4	6.87	57
Cingulate gyrus	32	R	8 22 34	6.96	173	10 18 30	7.22	156	2 6 44	8.73	204			
		L							−6 2 42	8.07	232	−4 2 50	6.40	34

#### Direct comparisons of TIA and PLAC groups

##### Extinction learning

**Figure 4:**
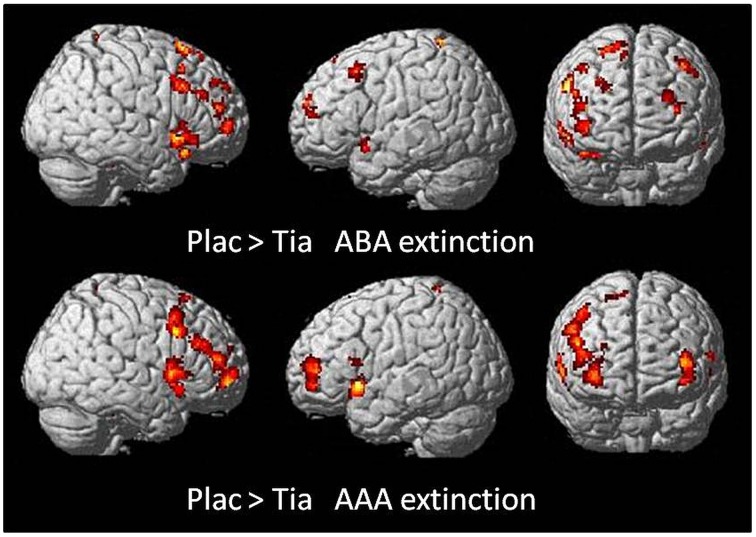
**Areas of reduced activation in the TIA group compared to the PLAC group in a two-sample ***t***-test (***p*** < 0.05 FWE-corrected on cluster level, minimum cluster size ***k*** = 10) for ABA and AAA extinction**. Activation in the TIA group is reduced predominantly in prefrontal regions, and also in further areas including hippocampus, insula and temporal pole.

A two-sample *t*-test showed reduced activation of the TIA group compared to PLAC during ABA and AAA extinction in bilateral dlPFC (BA 9) and OFC (BA 10), fusiform gyrus and temporal pole, as well as in right hippocampus and left lingual gyrus. Moreover, there was reduced activation in right lingual gyrus exclusively in ABA extinction, as well as reduced activation in left vmPFC (BA 10) and hippocampus, and in bilateral insula exclusively in AAA extinction (see Table 3 and Figure 4).

**Table 3:** **Two-sample test showing regions with higher activation in PLAC compared to TIA during extinction learning, ***p*** < 0.05 FWE-corrected, ***k*** = 10**.

**Brain region**	**BA**	**Hem**	**PLAC** > **TIA EXTINCTION ABA**			**PLAC > TIA EXTINCTION AAA**		
			**MNI x y z**	***t*-value**	**voxel**	**MNI x y z**	***t*-value**	**voxel**
Dorsolateral PFC	9	L	52 22 34	5.20	57	42 2 42	6.03	57
		R	22 56 32	4.78	37			
	8	L	−40 14 54	5.43	66			
	46	R				50 32 30	5.26	43
Orbitofrontal cortex	47	L	−28 22 −22	5.19	51	−45 15 −8	6.27	41
		R	52 20 −10	6.08	32	62 12 14	4.82	20
	10	L	−26 56 28	5.57	44	−42 52 4	6.14	41
		R	38 58 2	5.64	33	46 38 24	5.62	40
			44 48 18	5.20	53			
Ventromedial PFC	10	L				34 54 −4	6.26	80
Hippocampus		R	18 −32 −4	5.29	12	20 −26 −10	8.41	60
		L				−20 −30 −6	6.98	36
Fusiform gyrus	37	L	−36 −36 −24	5.96	89	−24 −48 −14	6.56	124
		R	36 −50 −16	5.32	135	32 −52 −14	6.57	92
Lingual gyrus		L	−18 −48 −10	4.13	29	−16 −48 −10		50
		R	20 −50 −6	5.09	26			
Insula	13	R				26 20 −16	5.64	20
		L				−26 20 −14	5.23	63
Temporal pole	38	L	−50 10 −12	4.70	34	−56 12 −2	5.10	54
		R	54 15 −12	4.12	29	52 16 −10	5.40	28

##### Recall

The two-sample *t*-test did not yield any significant activation differences between the groups in ABA and AAA recall.

#### Correlations between activation during ABA extinction learning and performance

Assuming that the reduced activation in extinction-relevant prefrontal and hippocampal regions in the TIA group was related to their learning performance, we performed across groups analyses correlating ABA extinction learning performance with brain activation in PFC and hippocampus during the task. Activation in PFC and OFC (mean activation of clusters in BA 8, 9, 10, 46; MNI coordinates 38 52 2, 44 48 18, 52 22 34, 38 58 2) showed a significant negative correlation with extinction learning performance (i.e., percent errors during ABA extinction learning), indicating that higher activation in these regions was associated with less errors in ABA extinction learning. (Pearson's *r* = −0.348 *p* = 0.016). Activation in a cluster comprising right-hemispheric hippocampus, MNI coordinates 18 -32 -4, too was negatively correlated with the number of extinction errors (Pearson's *r* = -0.286 *p* = 0.041).

## Discussion

In this study, we investigated the role of the dopaminergic system for extinction learning in changed and familiar contexts and for the renewal effect. While the DA-antagonist tiapride partially impaired extinction learning, it did not affect renewal *per se*. Associated with the TIA group's impaired extinction learning was a pattern of brain activation that, compared to the PLAC group, showed reduced activation in extinction-relevant brain areas.

### DA-antagonism impairs ABA extinction but not AAA extinction

According to our hypothesis, we observed extinction learning deficits in the DA-antagonist treated participants which were restricted to extinction learning in the presence of a novel context: in ABA extinction, TIA participants made significantly more errors than PLAC participants, while there was no significant difference in error rate between groups in AAA extinction learning. The TIA group's learning curves for ABA and AAA extinction show that while AAA extinction learning proceeded at a pace comparable to that of the PLAC group, in the ABA condition extinction learning was slowed down in both the early and late phases of learning. Moreover, the percentage of errors in ABA and AAA extinction differed significantly within the TIA group, but not within the PLAC group. The novelty of the context-cue compound in ABA presumably constituted a particular learning challenge for the TIA group which interfered with their learning progress.

The extinction deficit found in ABA extinction corresponds to studies in mice and rats in which manipulation of the dopaminergic system by D2 antagonists affected extinction learning (Ponnusamy et al., 2005; Mueller et al., 2010). Our results also correspond to findings from animal studies which recently demonstrated that local DA D1 or D2 antagonism in monkey prefrontal cortex impaired learning of novel associations while leaving recall of familiar associations intact (Puig and Miller, 2012; Puig et al., 2014). Our study extends these findings by showing that, in humans, D2/D3 receptor antagonism selectively impaired processing of a novel context-cue compound together with an altered outcome (ABA), while at the same time the manipulation had no adverse impact upon associating a changed outcome with a familiar context-cue compound (AAA).

### DA-antagonism does not affect renewal

In contrast to the findings for extinction learning, and contrary to our hypothesis, the selective impairment of ABA extinction learning in TIA participants did not affect the level of renewal. In both groups, a similar proportion of participants showed renewal. Furthermore, the REN participants of both groups showed a similar percentage of renewal effect responses in ABA recall, presumably due to the fact that also the TIA group eventually acquired the altered associations during extinction learning. This lack of a tiapride effect upon renewal is in line with findings reporting that recall of previously established associations is not affected by (D2) DA antagonism (Lee et al., 2007).

### Reduced prefrontal and hippocampal activation in extinction learning is associated with impaired ABA extinction

In parallel to the impairment of extinction learning in the ABA condition, the TIA group showed reduced BOLD activation in dlPFC and OFC during extinction learning. Moreover, the level of prefrontal activation was negatively correlated with learning performance across groups, with lower activation being associated with more errors in ABA extinction. These results are in line with findings from an animal study on associative learning in PFC which revealed a role for dopaminergic D1 and D2-receptors in modulating PFC-dependent learning (Puig et al., 2014; Puig and Miller, 2015). Antagonizing these receptor types impaired learning of new stimulus-response associations as well as cognitive flexibility, but not recall of familiar associations. Moreover, a recent fMRI study showed that DA signaling in human dlPFC was associated with encoding and updating of context information during a working memory task (D'Ardenne et al., 2012). Correspondingly, in our study, the reduced dlPFC activation in the TIA group was related to their deficits in ABA extinction learning, which required the integration of a novel context into an altered association between cue and outcome, an effort that was not necessary in AAA extinction learning. This interpretation is also in line with the findings of an fMRI study reporting a specific role for dlPFC in encoding relational information as opposed to item-specific information, indicating that dlPFC contributes to memory formation by building relationships between items (Blumenfeld et al., 2011).

Not only prefrontal, but also hippocampal activation reduction was correlated with more errors in ABA extinction. These findings correspond to previous research which showed that modulations of the dopaminergic system in hippocampus can affect learning and memory. In healthy humans, working memory-related dopamine release associated with D2 receptor availability was observed in hippocampus (Aalto et al., 2005). In addition, hippocampal D2 receptors were found to contribute to local functions such as long-term memory as well as to modulation of PFC functions, and thus might be involved in human executive function including working memory (Takahashi et al., 2007, 2008). D1 and D2 dopamine dependent negative feedback in the loop of hippocampus—basal ganglia- thalamus—hippocampus was shown to have a role in extinction of responses (Sil'kis, 2008). Given the role of hippocampus for context processing, our findings add to the existing evidence by suggesting that D2/D3 antagonism in hippocampus presumably affects processing of novel contexts during extinction learning.

### Further regions showing reduced activation associated with the DA-antagonist treatment

The lower activation observed in the TIA group in bilateral temporopolar regions may also have contributed to impaired extinction learning performance, since the temporal poles have been implicated in attentional processing (Lane et al., 1999), integration of semantic information (Noppeney and Price, 2002), object recognition (Nakamura and Kubota, 1996), and memory retrieval (Maguire et al., 2000). Furthermore, processes subserved by fusiform and lingual gyrus, such as visual encoding (Rombouts et al., 1999; McKenna et al., 2013), may have been compromised in the TIA group due to reduced activity in this region.

## Conclusion

In this study we investigated the role of dopamine for context-related associative extinction learning and renewal. Our findings for ABA extinction learning demonstrate a DA-antagonist related selective impairment in processing the combined load of an altered association together with a novel context, while changing an association between a cue and an outcome in a familiar context and subsequent renewal was not affected. Results suggest that in contextual extinction learning the dopaminergic system is specifically involved in readjusting the cue-outcome relationship in the presence of a novel context, with dopamine in PFC and hippocampus participating in this adjustment process. In contrast, relating context to the appropriate association and choosing the adequate response during extinction recall does not appear to exclusively rely on intact DA signaling.

## Funding

This work was supported by a grant from the DFG Deutsche Forschungsgemeinschaft (FOR 1581 Extinction Learning).

### Conflict of interest statement

The authors declare that the research was conducted in the absence of any commercial or financial relationships that could be construed as a potential conflict of interest.
